# An Algorithm to Classify Real-World Ambulatory Status From a Wearable Device Using Multimodal and Demographically Diverse Data: Validation Study

**DOI:** 10.2196/43726

**Published:** 2023-03-07

**Authors:** Sara Popham, Maximilien Burq, Erin E Rainaldi, Sooyoon Shin, Jessilyn Dunn, Ritu Kapur

**Affiliations:** 1 Verily Life Sciences South San Francisco, CA United States; 2 Department of Biomedical Engineering Duke University Durham, NC United States; 3 Department of Biostatistics & Bioinformatics Duke University Durham, NC United States; 4 Duke Clinical Research Institute Durham, NC United States

**Keywords:** digital measurement, wearable sensor, machine learning, ambulatory status, Project Baseline Health Study, physical activity

## Abstract

**Background:**

Measuring the amount of physical activity and its patterns using wearable sensor technology in real-world settings can provide critical insights into health status.

**Objective:**

This study’s aim was to develop and evaluate the analytical validity and transdemographic generalizability of an algorithm that classifies binary ambulatory status (yes or no) on the accelerometer signal from wrist-worn biometric monitoring technology.

**Methods:**

Biometric monitoring technology algorithm validation traditionally relies on large numbers of self-reported labels or on periods of high-resolution monitoring with reference devices. We used both methods on data collected from 2 distinct studies for algorithm training and testing, one with precise ground-truth labels from a reference device (n=75) and the second with participant-reported ground-truth labels from a more diverse, larger sample (n=1691); in total, we collected data from 16.7 million 10-second epochs. We trained a neural network on a combined data set and measured performance in multiple held-out testing data sets, overall and in demographically stratified subgroups.

**Results:**

The algorithm was accurate at classifying ambulatory status in 10-second epochs (area under the curve 0.938; 95% CI 0.921-0.958) and on daily aggregate metrics (daily mean absolute percentage error 18%; 95% CI 15%-20%) without significant performance differences across subgroups.

**Conclusions:**

Our algorithm can accurately classify ambulatory status with a wrist-worn device in real-world settings with generalizability across demographic subgroups. The validated algorithm can effectively quantify users’ walking activity and help researchers gain insights on users’ health status.

## Introduction

Quantifying physical activity can be highly informative about both general health status and the condition of people with specific diseases [1,2]. Characteristics of physical activity have been shown to be prognostic factors in various chronic conditions [3-13]. Yet reliably producing research-grade measurements of physical activity in real-world settings remains a challenge. Traditionally, the validation of such measurements often relies on individual self-reports or is performed episodically and in artificial laboratory environments. These approaches suffer from known challenges, such as subjectivity, assessment bias, and unreliability [14-16].

Recently, the advent of wearable technology has made it possible to measure physical activity to a previously untenable extent [17,18]. Ambulatory activity in particular, namely whether individuals are walking and how much, is a basic aspect of physical activity that can be investigated in general populations and in specific clinical settings. Wearable devices can collect information passively during daily living and generate a vast quantity of digital measurements that allow researchers to probe functional physical activity generally and ambulatory activity specifically. Using these digital measures in research studies, however, requires analytical validation [19]. In their design, validation studies have to balance factors such as feasibility and the resource-intensiveness of their data collection approach with demonstrating validity in representative populations.

To date, the majority of measurements in validation studies have come from either short observation periods in laboratory settings [20,21] or self-reported labels in real-world settings [22]. Laboratory measurements often render observations with exceptionally clean and easy-to-use ground-truth labels, but algorithms trained on data of this kind do not always generalize to everyday activities [23]. On the other hand, using self-reported labels as the ground truth yields a closer reflection of individual everyday activities, but these labels are often noisy and less accurate [15,16,24]. There have been some examples of reference devices deployed to generate accurate truth labels in generalizable real-world settings [25,26], but this came at the cost of intrusiveness and resource-intensive data processing steps after collection, such as manual video footage tagging. With all these considerations in mind, validation studies tend to be highly heterogeneous, and need to be interpreted in context.

Herein we report on the development and analytical validation of an ambulatory status classification algorithm. This algorithm classifies the ambulatory status of users of a wrist-worn device in real-world environments. We carried out 2 separate studies including participants from independent populations with distinct sources of ground-truth labels for a deeper characterization of the algorithm performance. One of the studies, the pilot program study, used a relatively small and demographically homogeneous cohort, where participants provided a highly accurate ground-truth source from a reference device. The other study was derived from the Project Baseline Health Study (PBHS), a prospective, multicenter, longitudinal study with participants of diverse backgrounds who were representative of the entire health spectrum [27]; this was a demographically diverse cohort that provided self-reported labels as the ground-truth source. This cohort was also relatively large, and we therefore expected it to yield results less susceptible to outlier readouts. We present analytical validation results of the performance of our algorithm against the highly accurate ground-truth source (from the pilot), and we examine the generalizability of the results across a study population of demographically diverse individuals (in the PBHS).

## Methods

### Participant Cohorts

Two distinct studies were conducted, with training and testing groups identified a priori within each study. Participants in both studies wore the smartwatch (the Verily Study Watch) [27-30].

The first study was a pilot program (n=75) of adult volunteer participants recruited among Verily Life Sciences employees in 2 locations (South San Francisco, California, and Cambridge, Massachusetts) without specific selection criteria. For this group, ground-truth labels were collected from an ankle-worn reference device (StepWatch 4). The Verily Study Watch and reference device were worn simultaneously for 7 consecutive days to ensure capture of both weekday and weekend behavior; for each participant, days were included as evaluable if both devices were worn synchronously for a minimum of 8 hours. No demographic information on race or ethnicity was collected in this study. The observation period ran from June to December 2019.

In order to expand the demographic representativeness of the overall validation effort, the second study included a large and diverse cohort (n=1691) consisting of participants from the PBHS who consented to participate in this substudy [27]. The period for data collection ran from May to December 2019.

### Ethics Approval

The pilot program was determined to be exempt research that did not require institutional review board review. Written informed consent was obtained from all participants enrolled in the PBHS; the PBHS was approved by both the WCG institutional review board (approval tracking number 20170163, work order number 1-1506365-1) and the institutional review boards of each participating institution (Stanford University, Duke University, and the California Health and Longevity Institute) [27]. The PBHS was registered at ClinicalTrials.gov (NCT03154346).

All methods complied with relevant guidelines and regulations; the research involving human participants was performed in accordance with relevant guidelines and regulations. Experimental protocols were approved by appropriate committees from Verily Life Sciences and by PBHS governance (participating institutions are above).

### Wearable Devices

**Figure 1:**
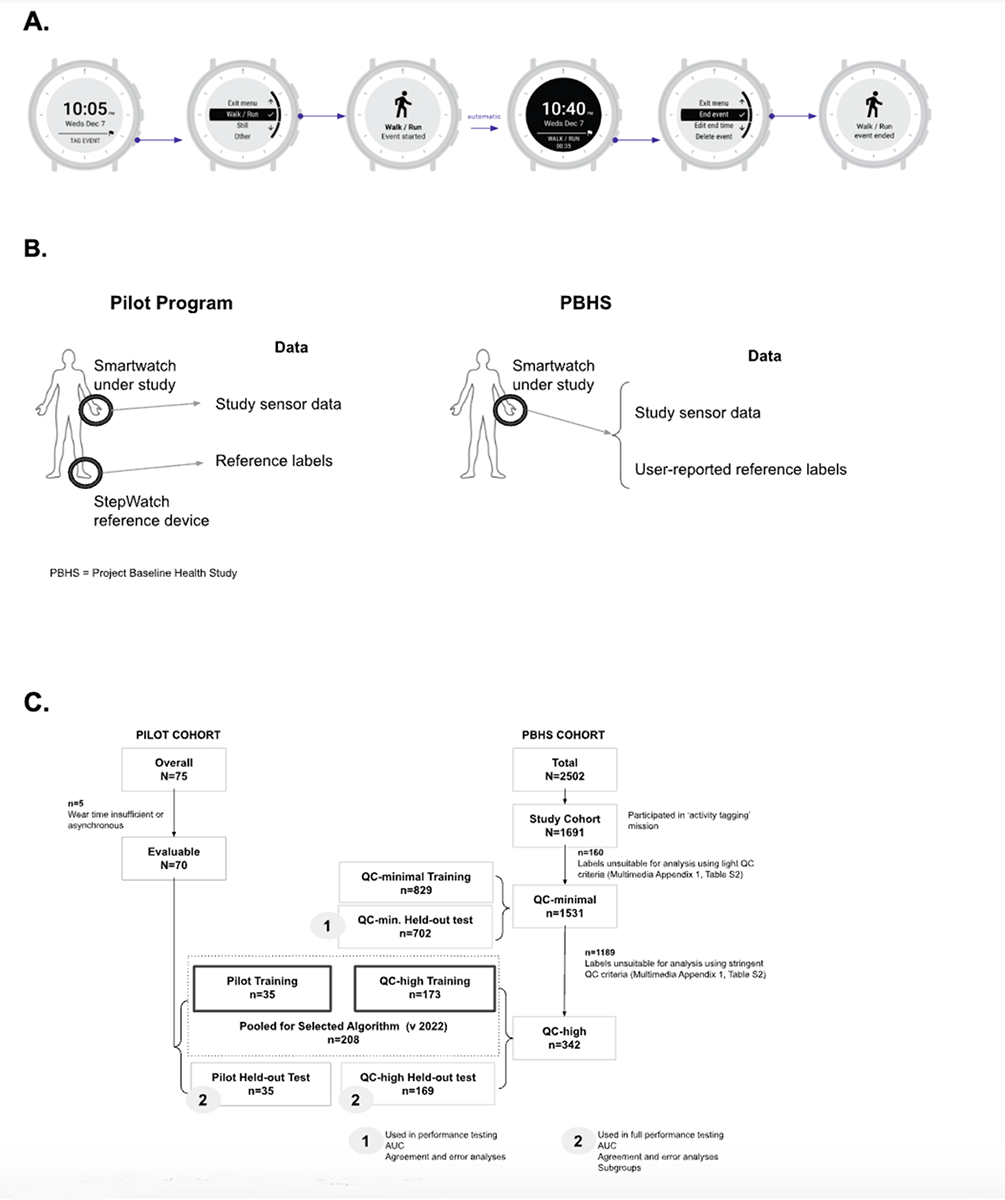
(A) Sketch of the user interface of the study device used in the Project Baseline Health Study. (B) Data elements for the 2 studies. (C) Flow of participant inclusion for the different cohorts and data sets in the 2 studies. AUC: area under the receiver operating characteristic curve; PBHS: Project Baseline Health Study; QC: quality control.

The Verily Study Watch recorded acceleration data in both cohorts via an onboard inertial measurement unit with a 30 Hz 3-axis accelerometer. For the PBHS population, the smartwatch also contained a user interface allowing participants to tag their activities on the watch (Figure 1A).

The reference device for the pilot program was an ankle-worn single-axis accelerometer (Modus StepWatch 4) that provided step count as a reference label for algorithm development.

### Reference Labels

In the pilot program, we generated reference labels on data collected from the ankle-worn StepWatch: 10-second windows were considered “ambulatory” if they had ≥3 steps on the wearing foot and “nonambulatory” if they had <3 steps [31]. The default window size returned by this device was 10 seconds, and this was deemed to provide good temporal granularity.

For the self-reported reference labels from the PBHS, participants tagged their activities as 1 of 3 options listed by the wrist device: “walk/run,” “still,” and “other.” Participants could tag the start and end of an activity period directly on the watch, which enabled precise synchronization of the labels to the raw sensor data stream. When necessary, participants could edit or delete tags as needed (Figure 1B). For the purpose of this analysis, “still” and “other” were grouped together under the “nonambulatory” label, while “walk/run” was equated to “ambulatory.”

The amount of data used from each of these studies is summarized in Multimedia Appendix 1, Table S3.

### Algorithm Development

Data from each study (pilot program, n=75; PBHS, n=1691) were split into nonoverlapping training and testing data sets at the participant level. For each study, data from approximately half the participants were used for training the algorithm and data from the other half were held out for algorithm testing. We decided on a 50-50 split in order to retain statistical power in the testing data, particularly considering the intended additional analyses of different demographic subgroups (discussed below).

In the pilot program, the split into training and testing data sets was based on participants’ daily step counts in order to mitigate potential algorithmic biases caused by training primarily on data from participants with either very low or very high activity levels. The difference in the mean daily step counts between the 2 halves of the split was 234 steps. For the PBHS cohort, the split into training and testing data sets was done randomly, as participants did not have daily aggregated results. We trained multiple versions of the algorithm with combinations of different subsets of training data and compared performance across these different algorithms (Figure 1).

We developed an algorithm that classifies the ambulatory status of device users in 10-second epochs (as ambulatory vs nonambulatory). First, the following 14 features were extracted from the Verily Study Watch’s acceleration data, in 10-second epochs: 3 features related to deviations of the signal, 5 features derived from power spectral density energy in frequency bands typically associated with user ambulation (ie, walking or running), 2 features that are signal percentiles (ie, 95th percentiles), and 4 features that are differences between signal percentiles (ie, IQR). These features were fed into a shallow neural network model with 2 dense layers: ReLu nonlinearities and softmax of outputs. The neural network was trained with a batch size of 32. The Adam optimizer was used with a learning rate of 0.001, and loss was calculated using categorical cross-entropy. Training ran for 10 epochs. Alternative features and neural network architectures were explored using the training data, but larger feature sets or more complex architectures did not result in higher performance, so this algorithm was chosen.

The classifier threshold was optimized to minimize absolute percentage error on daily ambulatory time on the training data from the pilot study (vs the data from the reference device used as the ground-truth source, as discussed above). For this optimization process, we performed 5-fold cross-validation at the participant level within the training data. We found the minimum daily mean absolute percentage error (MAPE) across the aggregated held-out data from all folds using a 1D grid search procedure.

The signal-processing, feature selection, model training, and hyperparameter tuning were all performed on training data sets identified a priori.

### Analyses

The demographic characteristics of the study cohorts were analyzed using descriptive statistics.

We analyzed the following metrics to characterize the performance of the algorithm, calculated on the held-out test sets: area under the receiver operating characteristic curve (AUC) for the overall study cohorts and across different demographic subcohorts within the PBHS cohort (this was chosen as the metric for comparison because, unlike other measures, such as *F*_1_-score or accuracy, it is not susceptible to differences in the chosen classifier threshold), mean accuracy, and MAPE of daily ambulatory time, defined as the summing of all 10-second windows that were labeled as “ambulatory” in a day.

Analyses were performed in python using *NumPy* (version 1.21.5), *pandas* (version 1.1.5), *SciPy* (version 1.2.1), *scikit-learn* (version 1.0.2), and *tensorflow* (version 2.10.0).

Confidence intervals were calculated using the bootstrap method with 1000 resampling iterations. Resampling was done at the participant level to ensure that all data from a single participant were either included or excluded within each resampling iteration.

## Results

### Characteristics of Participants From the Pilot Study and the PBHS Cohort

Participants in the pilot study were mostly male (45/75, 64%), with a mean age of 33 (SD 8.5) years. Participants from the PBHS were more often female (1366/2502, 55%), with a mean age of 54 (SD 17) years (Multimedia Appendix 1, Table S1).

### Algorithm Training

Data from each study were separately split (approximately 50-50) into nonoverlapping training and testing data sets (Figure 1); this allocation was done at the participant level (n=75 from the pilot study and n=1691 from the PBHS population). Out of 16.769 million 10-second epochs collected from the 2 studies, 8.841 million 10-second epochs were used for training across all algorithm iterations generated (the data sets are described in Multimedia Appendix 1, Table S3).

From the pilot program study, a total of 1,641,272 nonoverlapping 10-second epochs were collected (n=70 participants; Figure 1), of which 228,721 (13.9%) were “ambulatory” according to the reference device–based labels. We used 879,593 10-second epochs (from 35 unique participants) for training (118,730, 13.5% of which were “ambulatory”; Multimedia Appendix 1, Table S3).

We collected a total of 14,814,910 nonoverlapping 10-second epochs from the PBHS (n=1531 participants; Figure 1), of which 7,079,216 (47.8%) were “ambulatory” according to the participant-reported reference labels. The proportion of “ambulatory” labels in the PBHS was higher than in the pilot program study (47.8% vs 13.9%), which is likely attributable to the different labeling methods across studies. We expect that labeling from the pilot study was more stringent to show true ambulatory epochs, because these were determined directly by the reference device readouts (ie, any 10-second epoch with greater than or equal to 6 steps, relative to all 10-second epochs collected during the wear time). In the PBHS, the proportion of ambulatory labels was determined based on participant self-reported, manually entered walk/run tags relative to all entered tags. PBHS tagging, therefore, can be more vulnerable to selection bias toward “ambulatory,” since participants may favor reporting active over inactive states.

Data from the PBHS were not only divided into training and testing sets, but, across each set, we considered 2 quality control (QC) strata to test the impact of data quality on the development and performance of the algorithm. An extremely light QC selection, eliminating labels with gross apparent user errors (such as tags that were longer than a full day), was applied to generate the “QC-minimal” sub–data set, which therefore included virtually all labels suitable for evaluation (10,264/104,212, 9.8% of user-tagged events were eliminated, and another 12,010/104,212, 11.5% were truncated); a more stringent selection was applied to generate the “QC-high” sub–data set (80,852/104,212, 77.6% of user-tagged events were eliminated, and all tags were truncated to some degree; Figure 1 and Multimedia Appendix 1, Table S2). The 2 strata aimed to parse out performance variability due to noise generated by imperfectly self-reported reference labels (this was not a factor for the labels from the reference device in the pilot program). The resulting size of these QC training sub–data sets was 160,778 10-second epochs for QC-high (n=173 participants) and 7,802,829 for QC-minimal (n=829 participants). Of these labeled epochs, 102,783 (63.7%) and 3,863,964 (49.5%), respectively, were ambulatory according to the participant-reported tags (Figure 1 and Multimedia Appendix 1, Table S3).

### Effect of Raw Data Quality on Algorithm Performance

**Figure 2:**
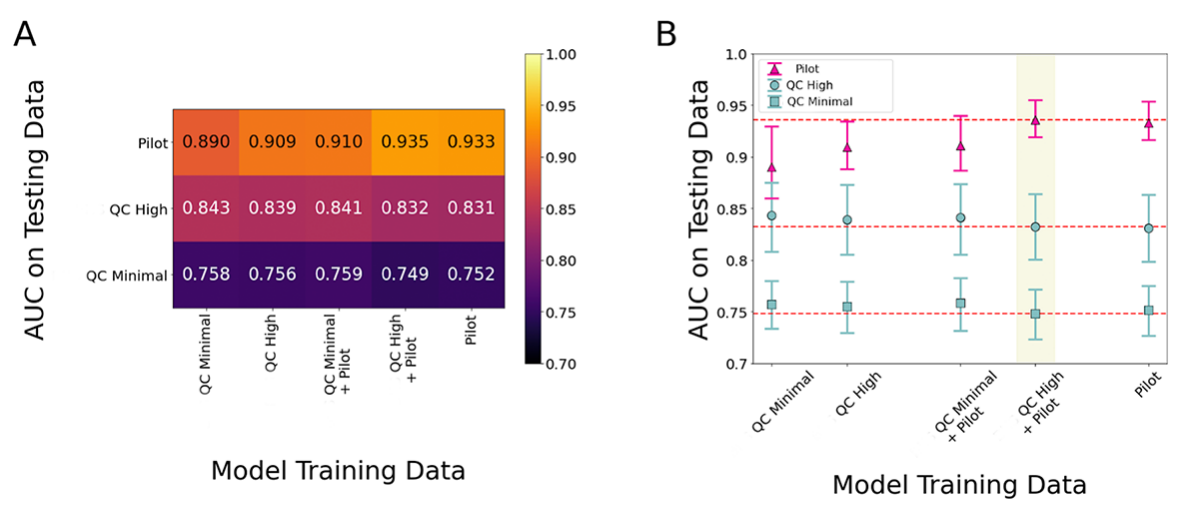
(A) Heat map of AUC values for the algorithm iterations generated via different training sub–data sets from the PBHS when tested on each of the separate testing cohorts. (B) AUC values for the algorithm iterations generated via different training sub–data sets from the PBHS when tested on each of the separate testing cohorts, with error bars based on the 95% CI. Each testing cohort is shown with a different color or symbol. From top to bottom, the red dotted lines indicate mean AUC values for the pilot, PBHS QC-high, and PBHS QC-minimal test data sets, respectively. The model trained on combined PBHS QC-high and pilot training data (highlighted in yellow) was the version of the algorithm used for further analyses. AUC: area under the receiver operating characteristic curve; PBHS: Project Baseline Health Study; QC: quality control.

We tested each of the algorithm iterations from the training process above (originated using the 2 PBHS QC sub–data sets and the pilot data set) across data from the held-out QC sub–data sets from the PBHS and the pilot program by calculating AUC values across all combinations. Namely, we tested each of the following algorithms against the held-out data sets from the pilot study and the PBHS QC-high and QC-minimal sub–data sets (Figure 2): (1) trained with the PBHS QC-high sub–data set, (2) trained with the PBHS QC-minimal sub–data set, (3) trained with the pooled PBHS QC-high plus pilot data set, (4) trained with the pooled PBHS QC-minimal plus pilot data set, and (5) trained with just the pilot data set. For each algorithm iteration, AUC values varied across the testing sub–data sets (QC sub–data sets from the PBHS and pilot program), with differences ranging from 0.047 to 0.187. For each test data set, the AUC variations across the algorithm iterations (1) through (5) were narrower, with differences ranging between 0.001 and 0.045. Therefore, data quality differences across the training sub–data sets did not appear to affect algorithm performance, as reflected in AUC variability, as much as data quality in the testing sub–data sets.

Based on the testing results described above, we selected an algorithm trained using combined data from one of the PBHS sub–data sets (QC-high) plus the pilot program data set to proceed to further analysis. This algorithm iteration (termed “version 2022”) showed the highest testing performance (evaluated by AUC) calculated with data from the pilot program (the most precise and cleanest data set) without substantially reduced performance on PBHS data (Figure 2). With this approach, we prioritized testing the accuracy of the algorithm against participants’ actual ambulatory status based on the reference device, not against the type of labels that are most feasible to obtain (ie, self-reported labels), although we report accuracy on both types of labels.

### Algorithm Testing

**Figure 3:**
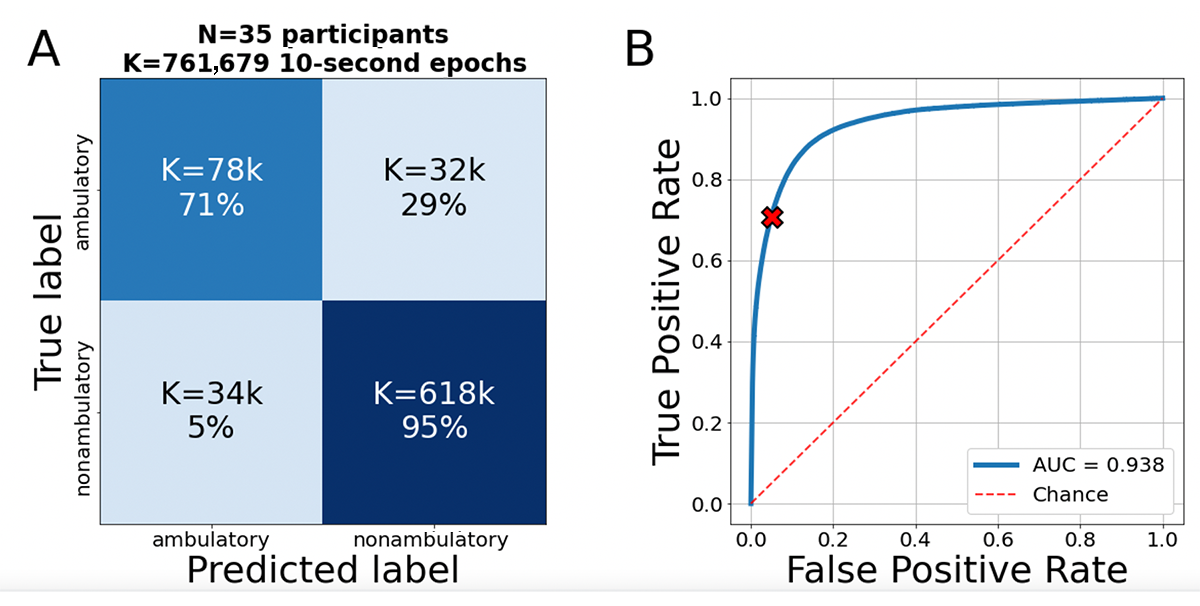
(A) Accuracy of the algorithm selected for full analysis, as evaluated in the pilot cohort. Here, the color map denotes K, the number of 10-second epochs. Percentages are normalized across rows, which allows easy reading of the sensitivity and specificity values. (B) Receiver operating characteristic curve and area under the curve of the algorithm selected for full validation, as evaluated in the pilot cohort. The red X denotes the true positive rate and false positive rate of the algorithm at the chosen classifier threshold. AUC: area under the receiver operating characteristic curve.

Tested against the held-out data set from the pilot program (Table 1), the selected algorithm had a sensitivity of 71% and a specificity of 95%, for an overall accuracy of 91.5% (95% CI 90.3%-92.9%; Figure 3A) and an AUC of 0.938 (95% CI 0.921-0.958; Figure 3B) when classifying the ambulatory status of 10-second epochs. When tested on the held-out data set from the PBHS QC-high sub–data set, the selected algorithm had an overall accuracy of 75.7% (95% CI 72.5%-78.6%) and an AUC of 0.832 (95% CI 0.800-0.864).

**Table 1:** Algorithm performance measures.

	Accuracy	Sensitivity	Specificity	PPV^a^	*F*_1_-score	AUC-ROC^b^	AUC-PRC^c^
Pilot study	91.3%	0.706	0.948	0.696	0.701	0.938	0.781
PBHS^d^ QC^e^-high	75.8%	0.731	0.802	0.885	0.788	0.832	0.901

^a^PPV: positive predictive value.

^b^AUC-ROC: area under the receiver operating characteristic curve.

^c^AUC-PRC: area under the precision-recall curve.

^d^PBHS: Project Baseline Health Study.

^e^QC: quality control.

**Figure 4:**
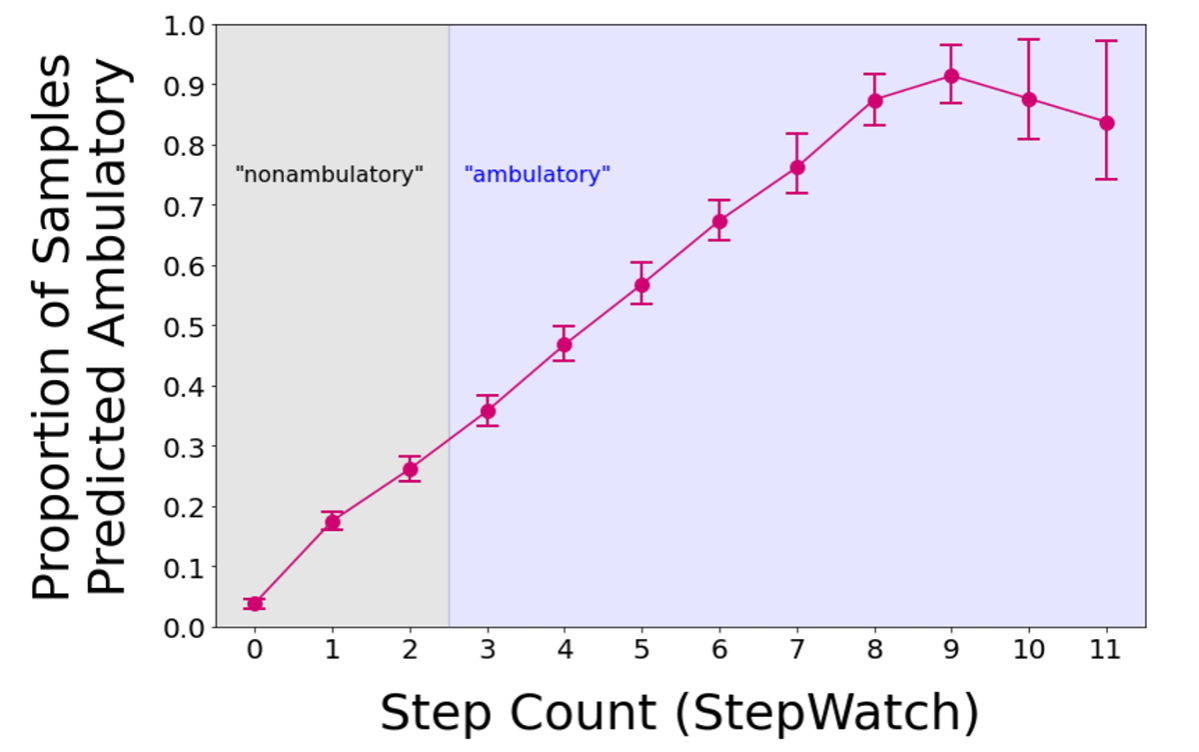
Predictions of the selected algorithm to classify 10-second epochs as ambulatory (or not) according to the number of steps in the 10-second epochs based on the reference device data from the pilot program study. A perfectly performing algorithm would predict “ambulatory” for all epochs with 3 or more steps on the wearing foot (indicated by the blue shadow), and nonambulatory for all epochs with fewer steps (indicated by the gray shadow). Epochs with more than 11 recorded steps are not shown due to their small sample size (Multimedia Appendix 1, Figure S1).

The proportion of predicted ambulatory epochs of the selected algorithm varied with the number of steps in the 10-second epochs (Figure 4). The lowest proportion of predicted ambulatory epochs happened in the 3 to 5 step range (36%-57% sensitivity, ie, correct predictions as “ambulatory: yes”), and the proportion of epochs classified as ambulatory grew with additional steps in the 10-second window (67%-91% correct predictions). Note that data from epochs with more than 11 recorded single-leg steps are not shown due to their low frequency (the number of samples per step count is shown in Multimedia Appendix 1, Figure S1).

**Figure 5:**
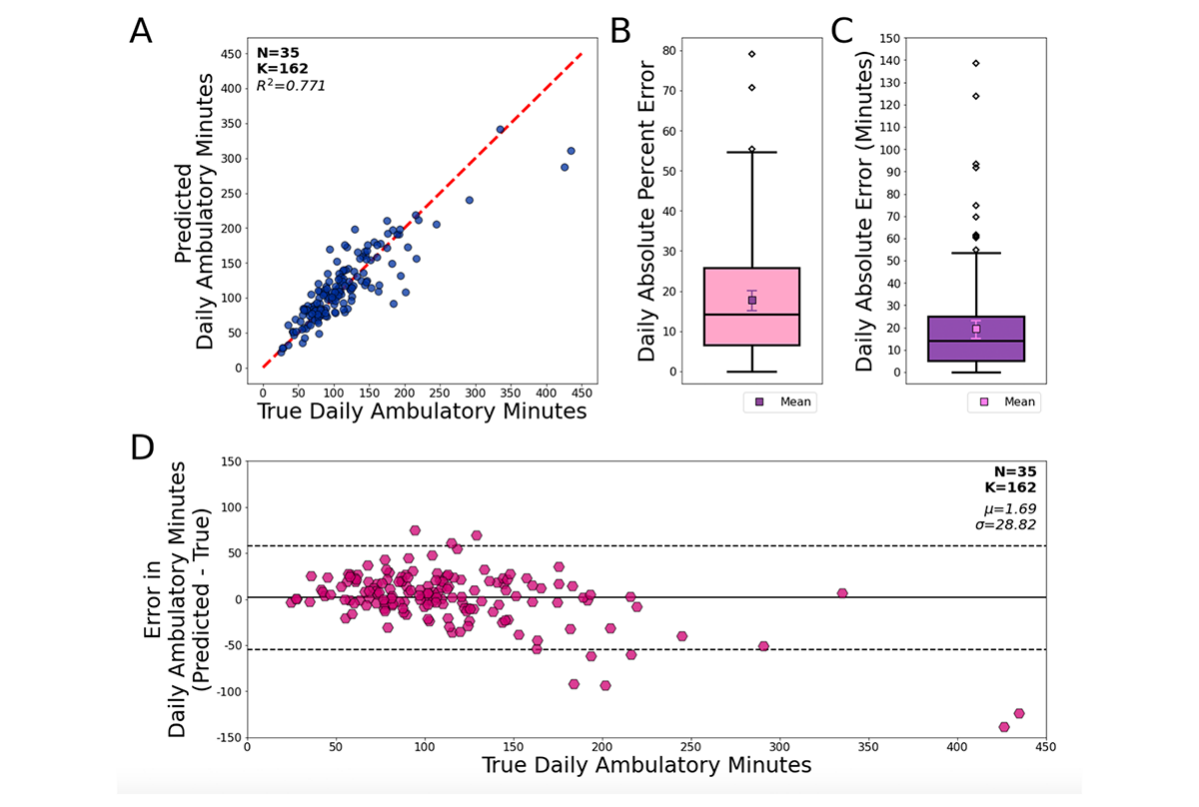
Agreement and error rates of the algorithm predictions. K is the number of user-days. (A) Agreement between the selected algorithm’s predictions and the ground-truth source for daily ambulatory time in the pilot-testing data set. (B) Absolute percent error in daily ambulatory time: median (pink box) and mean (purple box). (C) Absolute error in daily ambulatory time in minutes: median (purple box) and mean (pink box). (D) Modified Bland-Altman plot showing error in daily ambulatory time (in minutes) as it relates to the ground-truth daily ambulatory time.

When considering daily step aggregates as the metric of interest, there was good agreement between the algorithm classifications and the reference (*R*^2^=0.771), with a MAPE in daily ambulatory time (minutes) of 18% (95% CI 15%-20%) and a median absolute percentage error of 14% (Figure 5A and Figure 5B). The mean absolute error (MAE) of daily ambulatory time was 19.5 (95% CI 15.0-23.2) minutes, and the median absolute error was 14 minutes (Figure 5C). Consistent with the observations at the 10-second epoch level, the magnitude of error in daily ambulatory time (ie, the difference between algorithm-predicted and actual values) was dependent on the actual daily ambulatory time (as computed by the StepWatch; Figure 5D): the chance for underestimating daily ambulatory time (in minutes) grew as the reference daily ambulatory time increased. The largest underestimation we observed was 138.5 minutes in absolute time (relative error 32.5%).

### Performance of the Ambulatory Status Classification Algorithm Across Demographic Subgroups

**Figure 6:**
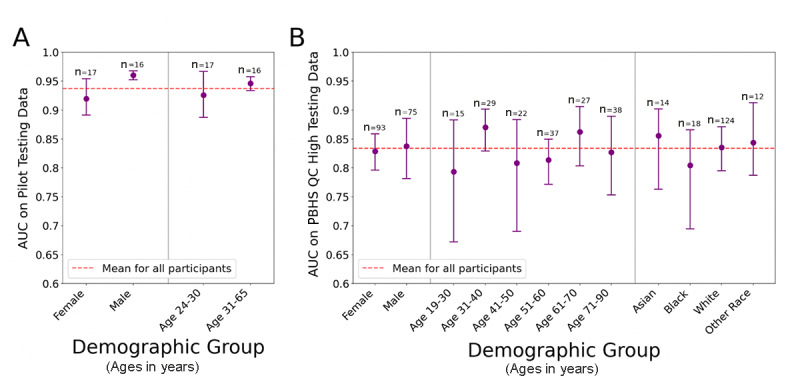
Performance (AUC values) of the selected algorithm across different demographic subgroups. (A) The pilot study testing data set. (B) The PBHS QC-high testing data set. AUC: area under the receiver operating characteristic curve; PBHS: Project Baseline Health Study; QC: quality control.

In order to characterize the generalizability of the algorithm’s performance, we calculated AUC values for the selected algorithm across demographic subgroups based on gender, age, and race. Initially, in the testing data set from the pilot program (Figure 6A), the results suggested a possible difference in performance between male and female participants, as seen in the lack of overlap of the 95% CIs. However, in a similar analysis using the larger and more diverse testing data set from the PBHS, which enabled subanalyses by participant gender, race, and age, that difference was no longer present and the results showed no meaningful performance difference across any of the subgroups of age, gender, or race, as evidenced by the overlapping 95% CIs (Figure 6B). A replication of the majority population from the pilot study within the PBHS showed an AUC of 0.8166 (95% CI 0.7501-0.8666) for White males aged 31 to 65 years in the PBHS cohort, which was not significantly different from the AUC of the PBHS cohort as a whole (AUC 0.8339).

## Discussion

This study presents the analytical validation in a real-world setting of an algorithm to classify the ambulatory status of users wearing a smartwatch. The algorithm performs well, distinguishing between ambulatory and nonambulatory states with high accuracy (75.7%-91.5% depending on the testing data set). Furthermore, the approach taken to analytic validation allowed us to investigate multiple subgroups, including age, gender, and race, demonstrating that the high performance of the algorithm is generalizable across a broad range of demographics.

All existing validation studies of ambulatory status classification from wrist-worn sensors have been either performed on young and healthy populations [25] or in the laboratory or clinic [20,21]. Yet measuring ambulatory status or daily ambulatory time is most clinically relevant for people with walking impairments—whether due to age, movement disorders, cardiovascular illness, or other circumstances—and most informative when done in an individual’s own environment (ie, their real-world setting). Thus, a key innovation in this work is our focus on using data captured in real-world settings (as opposed to highly controlled clinic or laboratory settings) from demographically diverse cohorts for the actual development and validation of this algorithm.

Therefore, the novel contributions of this work are 2-fold. First, we introduce a scalable framework for collecting reference labels on ambulatory status via a reference device and via user-reported data for training and validation. As part of that approach, we used 2 separate and different modalities to measure ground-truth status. This strategy enabled us to handle both comprehensive and highly precise labels (in the pilot program), as well as a larger volume of inherently noisy ones (user-reported tags from the PBHS), both in real-world settings. Our strong results across both sets of data indicate that this innovative multimodal approach contributed to a robust development scheme that may have boosted the performance of the resulting algorithm. The long-term practical convenience of a wrist-worn device (as opposed to an ankle-worn device or a dedicated assessment period) may be advantageous for this type of continuous generalizable monitoring [32-35], although a thorough side-by-side analysis of these 2 reference standard measurement methods to fully understand their correlation remains as a topic for future studies.

Second, we leveraged this framework to provide large-scale validation of the performance of the selected algorithm iteration, addressing shortcomings in terms of generalization previously reported in the literature [20,21,32]. Prior studies have used algorithms to report on differences in physical activity by different demographic subgroups but lacked validation data for those algorithms across demographic subgroups [25,36-38]. To our knowledge, this is one of the first studies to show a proper validation approach to develop and test a generalizable algorithm across demographic subgroups where algorithm output could have differed by subgroup.

In addition, our approach highlights several points of interest when developing validation methodologies for this type of algorithm. The increased sample sizes and variability in data quality achieved by combining 2 distinct data sets enabled deeper characterization of the algorithm’s performance. One of our studies generated data sets where truth labels were of high quality and accuracy but were collected from a study population limited in scope; the other study collected data from a large and demographically diverse cohort (albeit a somewhat engaged and self-selected participant group who volunteered and expressed interest in the PBHS and its health technology aspects), which allowed us to conduct subgroup analyses for both training and testing. Our results reinforce the well-established fact that modern machine-learning algorithms can sometimes perform well even when trained on a noisy data set [39]. This observation may be useful for researchers navigating study design decisions and tradeoffs, including sample sizes and data labeling methods. For future research, determining the role of data quality factors in the development and characterization of this type of algorithm is an open issue [18].

Our approach to the generation of reference labels was pragmatic, using deployment-friendly ankle-worn devices or user-reported tags. Neither of these was as resource-demanding as other intensive approaches (ie, video monitoring), but generated information of sufficient quality to conduct our validation with relatively high time resolution (10-second epochs). Of note, the intrinsic nature of the 2 methods used for the generation of reference labels probably contributed to the noticeable difference in the proportions of “ambulatory” labels between the 2 studies (discussed in the Results), with the proportion observed in the pilot program study being the one closest to other literature reports [40].

When interpreting our results in the context of existing literature, it is worth noting that most validation studies for this type of algorithm have used step counts as the metric of interest [31,36,41-53], while ambulatory time (or a related metric) is the focus of a minority of reports [54,55]. In general, considering the close correlation between step count and ambulatory time, the performance of our algorithm could be placed on par with other algorithms, yet detailed side-by-side appraisals of results remain challenging; this research field is in need of standardization [19,56,57].

This study also had limitations. First, in principle, the StepWatch readouts used as ground truth may not have provided perfect accuracy, even though there is extensive literature supporting the use of StepWatch as a reference device [31,50,51,56,58-60]. Second, we observed fluctuations in the ambulatory status classification algorithm performance based on daily ambulatory time; this fluctuation was present when the algorithm detected 10-second epochs as ambulatory (or not) and was also manifested in the daily aggregates of ambulatory time. While this trend (shown in Figure 5) may have been driven, partially, by outlying data points with high step counts in our sample, which would be of little relevance in hypothetical clinical scenarios, it may also have been due to low-step periods containing mixed activities in which walking was not the only or dominant source of hand motion. In addition, while the cutoff used to read the StepWatch ambulatory classification relied on existing literature [61], it may not be perfect in itself. In this regard, it could be reassuring that the algorithm handled epochs with step counts between 4 and 8 as a continuum, as this is possibly reflective of the complexities of organic movement.

In sum, we have developed an accurate algorithm for the detection of the ambulatory status of users of a wrist-worn device in a free-living, real-world setting; the output is generalizable across several user demographic characteristics. The characterization of this algorithm was conducted in 2 distinct data sets, which lends credibility to the robustness and applicability of the performance results obtained in this study and illustrates the advantages of similar approaches to future research in this field.

## Appendix

Multimedia Appendix 1 Supplementary materials.

## Abbreviations

AUC: area under the receiver operating characteristic curve
IRB: institutional review board
MAE: mean absolute error
MAPE: mean absolute percentage error
PBHS: Project Baseline Health Study
PPV: positive predictive value
PRC: precision-recall curve
QC: quality control
ROC: receiver operating characteristic

## Data Availability

The deidentified PBHS data corresponding to this study are available upon request for the purpose of examining their reproducibility. Interested investigators should direct requests to jsaiz@verily.com. Requests are subject to approval by the Project Baseline Health Study governance. Data from the pilot program are not available due to the nature of this program. Participants in this program did not consent for their data to be shared publicly.
