# Supplementary material for: An Algorithm to Classify Real-World Ambulatory Status From a Wearable Device Using Multimodal and Demographically Diverse Data: Validation Study
Source: JMIR Biomed Eng. 2023 Mar 7;8:e43726. doi: 10.2196/43726 (PMC11041455; doi:10.2196/43726)
Supplement: Multimedia Appendix 1 [file biomedeng_v8i1e43726_app1.docx]

## **SUPPLEMENTARY MATERIALS**

**Validating an algorithm to classify real-world ambulatory status from a wearable device using multimodal and demographically diverse data**

Sara F. Popham, PhD; Maximilien Burq, PhD; Erin Rainaldi; Sooyoon Shin, PhD;
Jessilyn Dunn, PhD; Ritu Kapur, PhD.

**Supplementary Figure 1.** Amount of data in the Pilot Study, binned by step count returned by the reference device. Data for 12 and 13 steps within a 10-second window are not shown in Figure 4 due to the low number of samples (483 and 8 samples, respectively).

**
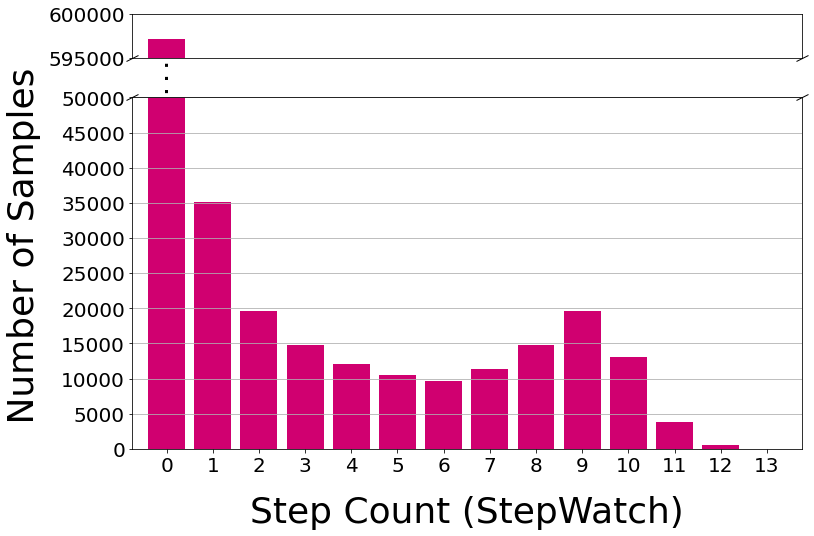
**

**Supplementary Table 1. Participant characteristics across cohorts**

|  |  | **Pilot Study**  **Cohort**  **N=75** | **PBHS** | | |
| --- | --- | --- | --- | --- | --- |
|  |  |  | **QC-high Test Subset**  **n=169** | **Study Cohort**  **N=1691** | **Total**  **N=2502** |
| **Sex,  n (%)** | **Female** | 25 (35.7) | 93 (55.4) | 922 (55.0) | 1366 (55.0) |
|  | **Male** | 45 (64.3) | 75 (44.6) | 753 (45.0) | 1117 (45.0) |
| **Race,  n (%)** | **Asian** | n/a | 14 (8.3) | 170 (10.1) | 260 (10.4) |
|  | **Black** | n/a | 18 (10.7) | 226 (13.5) | 397 (15.9) |
|  | **White** | n/a | 124 (73.8) | 1128 (67.3) | 1568 (62.7) |
|  | **Other** | n/a | 12 (7.1) | 151 (9.0) | 258 (10.3) |
| **Age, yrs** | **Median** | 30 | 55 | 55 | 50 |
|  | **Mean (SD)** | 33.2 (8.5) | 54.3 (17.1) | 54.1 (16.8) | 50.5 (17.1) |
|  | **Range** | 23-63 | 19-87 | 19-92 | 19-92 |
| Race information was not collected (not available, n/a) from the Pilot Study.  PBHS=Project Baseline Health Study; QC=quality control; SD=standard deviation | | | | | |

**Supplementary Table 2. Criteria applied to label tags to define the different data-quality strata from the BHS cohort, selected strata.**Note that the criteria are not mutually exclusive: the QC-high is a subset of data in the QC minimal cohort

| **QC Level** | **Min Length** | **Max Length** | **Proportion of epochs kept from each single tagged event*** | **Proportion of epochs kept from each edited tagged event**** | **Discard first __ tags (or entire 1st day if fewer)** | **Training Required?** | **Discard tags longer than** | **Removed time from onset of event tagging** |
| --- | --- | --- | --- | --- | --- | --- | --- | --- |
| **Minimal** | 15 s | 3 h | 100% | First 95% | 0 | No | 10 h | None |
| **High** | 30 s | 5 min | First 50% | First 50% | 5 | Yes | 2 h | 15 s |

*For example, for High QC level, tags from the first 50% of epochs during a single tagged event were kept.
** For example, for Minimal QC level, tags from the first 95% epochs during single, *edited* tagged events were kept.

**Supplementary Table 3. Data within each QC level from the PBHS study**

|  |  | **Participants** | **User-tagged events** | **10-second epochs** | **Proportion of Ambulatory Time** |
| --- | --- | --- | --- | --- | --- |
| **QC-high** | **Training** | n=173 | E=11,978 | K=160,778 | 63.7% |
|  | **Testing** | n=169 | E=11,382 | K=151,557 | 61.3% |
| **QC-minimal** | **Training** | n=829 | E=47,632 | K=7,802,829 | 49.5% |
|  | **Testing** | n=702 | E=46,316 | K=7,012,081 | 45.9% |
| **Pilot cohort** | **Training** | n=35 | NA | K=879,593 | 13.5% |
|  | **Testing** | n=35 | NA | K=761,679 | 14.4% |
| NA=not applicable; QC=quality control | | | | | |
